# Overkilling in a Dog: A Case Report

**DOI:** 10.3390/ani15060884

**Published:** 2025-03-19

**Authors:** Federica Pesce, Emanuela Sannino, Enza Ragosta, Laura Marigliano, Giuseppe Picazio, Mauro Esposito, Maria Dimatteo, Barbara Degli Uberti, Susanna De Luca, Noemi Di Caprio, Domenico Citarella, Renato Pinto, Giovanna Fusco, Esterina De Carlo, Gianluca Miletti

**Affiliations:** 1 Department of Animal Health, Istituto Zooprofilattico Sperimentale del Mezzogiorno, 80055 Portici, Italy; federica.pesce@izsmportici.it (F.P.); enza.ragosta@izsmportici.it (E.R.); maria.dimatteo@izsmportici.it (M.D.); barbara.degliuberti@izsmportici.it (B.D.U.); giovanna.fusco@izsmportici.it (G.F.); gianluca.miletti@izsmportici.it (G.M.); 2 Department of Chemistry, Istituto Zooprofilattico Sperimentale del Mezzogiorno, 80055 Portici, Italy; laura.marigliano@izsmportici.it (L.M.); giuseppe.picazio@izsmportici.it (G.P.); mauro.esposito@izsmportici.it (M.E.); 3 Prevention Department Asl Napoli 3 Sud, 80053 Castellammare di Stabia, Italy; s.deluca@aslnapoli3sud.it (S.D.L.); n.dicaprio@aslnapoli3sud.it (N.D.C.); d.citarella@aslnapoli3sud.it (D.C.); r.pinto@aslnapoli3sud.it (R.P.); 4 National Reference Centre for Hygiene and Technologies of Water Buffalo Farming and Productions, Istituto Zooprofilattico Sperimentale del Mezzogiorno, 84131 Salerno, Italy

**Keywords:** overkilling, asphyxia, toxicology

## Abstract

The term “overkilling” in forensic medicine is not clearly defined and is used to refer to homicides involving unusually massive injuries, far exceeding those necessary to kill the victim. This is the clinical case of a dog found in February 2023 in a town near Naples, with a rope around its neck and the metacarpal region of its forelimbs. The dog was taken to the Istituto Zooprofilattico Sperimentale del Mezzogiorno (IZSM, Portici, Southern Italy), where it underwent a total body radiographic study performed using the “Philosophy HF400” X-ray device (Pan Vet, Kildare Town, Ireland). Subsequently, a full autopsy was performed, and samples of the injured organs were analyzed by accredited in-house laboratories for microbiological, histological and toxicological analyses. The autopsy revealed anatomopathological lesions compatible with strangulation, which were confirmed by histological examination. The autopsy also reported serosanguineous chest effusion, food material mixed with blackish microgranules and harmful substances in the stomach and, finally, uncoagulated blood in the atrioventricular chambers of the heart. These findings raised the suspicion of poisoning, which was confirmed by the positive outcome of toxicological tests.

## Simple Summary

This case report describes the investigation of a young large-breed dog found dead with signs of severe abuse, including a rope around its neck and legs. This study aimed to uncover the cause of death and related events. The examination revealed multiple injuries, including bruising, internal bleeding and a fractured neck bone, suggesting strangulation. X-ray images showed unusual materials in the stomach, while laboratory tests detected poisons commonly used to kill rodents in the dog’s liver. These findings indicate that the dog had been subjected to both physical abuse and poisoning. This report highlights the importance of forensic veterinary science in identifying cases of animal cruelty and ensuring justice, not only by protecting animals but also by raising awareness of how violence towards animals can be linked to violence against people. Veterinarians play a vital role in protecting both animal welfare and public safety.

## 1. Introduction

The term “overkilling”, rarely used in forensic literature, refers to a specific type of homicide characterized by an excessive number of wounds inflicted on the victim, far beyond those necessary to cause death [1]. It is often associated with murders characterized by extreme and repeated violence and is also used in other fields, such as antibiotic therapies or radical surgery, to underline the exaggeration of a treatment and even in reference to weapons of mass destruction.

There is no universally accepted definition of “overkill” based on the experience of professionals who perform autopsies and scholars who conduct research. However, there are two basic criteria for classifying a case as “overkill”: it must be a homicide, and the number of wounds must exceed that necessary to cause death. However, even a single wound can be classified as “overkill” in cases of extreme brutality [2].

The types of injuries considered include sharp instrument wounds (cut wounds, puncture wounds), blunt object wounds (internal trauma or external injuries) and gunshot wounds. In addition, strangulation or combined killings with different methods are also included [2,3].

In veterinary medicine, overkill is a rather rare phenomenon that must be examined in the most detailed way: forensic doctors must evaluate not only the number of wounds but also their distribution and morphology and the weapon used to inflict them [3].

Although often criticized as merely anecdotal, evidence supporting a connection between animal abuse and violence toward humans has grown over the years [4]. In 1994, Arkow stated that veterinarians have a crucial role to fulfill, as they are the ones who observe and care for abused animals. Moreover, he believed that veterinarians not only have an ethical duty towards animals but also a broader societal responsibility “to take a leading role in preventing abusive interactions” [5].

In 1983, Hutton proposed that evidence of abuse (of any kind) toward a family pet could serve as valuable information for the early identification of abuse in other family members [4]. However, to use animal abuse as an indicator, it is essential to be able to recognize and identify such abuse. This may seem simple in cases of neglect.

The forensic autopsy approach is the first step in a solid forensic investigation that leaves no stone unturned and leads to convictions for those involved. However, the diagnosis should be based on a combination of solid evidence, a detailed inspection of the location where the body was discovered, the exclusion of other potential medical conditions and the identification of consistent injuries through a meticulous autopsy [6].

## 2. Materials and Methods

**Figure 1:**
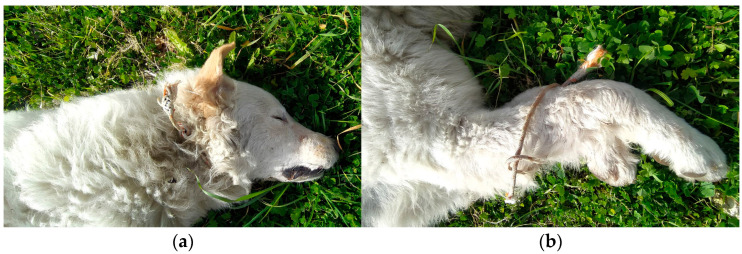
Dog found dead with a rope around its neck (**a**) and one around the metacarpal region of the right and left forelimbs (**b**).

On 7 February 2023, in a town near Naples, a giant-sized male dog of about 2 years old was found dead with a rope around his neck and one around the metacarpal region of his right and left front limbs (Figure 1).

On the same day, the dog was taken to the Istituto Zooprofilattico Sperimentale del Mezzogiorno (IZSM, Portici, Italy), where it underwent a total body radiographic examination carried out using the “Philosophy HF400” radiographic device (Pan Vet, Kildare Town, Ireland) in right latero-lateral and ventro-dorsal projections.

A forensic autopsy was subsequently performed following the National Guidelines for Forensic Autopsies in Veterinary Medicine [6]. At the end of the autopsy, samples for microbiological, histological and toxicological analyses were collected.

Specifically, for microbiological analysis, approximately 20 mg of tissue was collected for each organ using sterile forceps and scissors and then transferred separately into 2 mL test tubes.

Furthermore, fragments of damaged and normal skin, trachea and hyoid bone were collected and fixed in 10% neutral buffered formalin for histological examination. Hyoid bone was decalcified. Tissue samples were trimmed and embedded in paraffin. Microsections 3–4 μm thick were routinely stained with hematoxylin and eosin with a standard protocol (Leica Autostainer XL, Leica Biosystems, Deer Park, IL 60010, USA).

Finally, gastric contents and liver were sampled for toxicological analyses, performed on the liver for the detection of rodenticides by liquid chromatography–mass spectrometry (LC–MS/MS) and on the gastric contents for the detection of pesticides by gas chromatography–mass spectrometry (GC–EI/MS).

The analysis of anticoagulant rodenticides (bromadiolone, brodifacoum, coumachlor, coumatetralyl, difenacoum and warfarin) was performed following an internal method, a modification of one already described [7]. The reference standards for six rodenticides were obtained from Dr. Ehrenstorfer Gmbh. Confirmation of the identity of the compounds was performed by acquiring two transitions in MRM (multiple reaction monitoring) mode to have a qualifier ion and a quantifier ion. The detailed parameters of 6 rodenticides, including precursors and product ions, are given in Table 1.

The method adopted is qualitative confirmatory, although a limit of quantification was determined at the lowest level of the validation experiments (LOQ = 0.5 mg kg^−1^) according to the following criteria: recovery between 80 and 120% and RSD < 20%. The linearity of the method was confirmed by the correlation coefficient values (R^2^ > 0.99) obtained for the calibration curve set for all six analytes with reference material solutions (0.010–0.500 mg L^−1^).

The analytical methodology used for the detection of organochlorine, carbamate, pyrethroid and organophosphorus pesticides combines conventional solid–liquid extraction of the pesticides from the sample, purification and analysis by gas chromatography–mass spectrometry (GC–EI/MS) [8].

The organochlorine pesticide solution contains Alpha-HCH, Beta-HCH, Gamma-HCH (Lindane), Aldrin, Dieldrin, Endosulfan-alpha, Endosulfan-beta, Heptachlor, Heptachlor-trans epoxide, Heptachlor-cis epoxide Endrin, cis-Chlordane, trans-Chlordane, Hexachlorobenzene, Dichlorodiphenyltrichloroethane and its metabolites (DDT).

The carbamate and pyrethroid pesticide solution contains Carbaryl, Propoxur, Cypermethrin, Permethrin, Bendiocarb, Bifenthrin, Carbofuran, Methiocarb and Propham.

The organophosphorus pesticide solution contains Azinphos-ethyl, Azinphos-methyl, Bromophos-ethyl, Bromophos-methyl, Chlorpyrifos, Chlorpyrifos methyl, Diazinon, Dimethoate, Ethion, Fenchlorphos, Malathion, Methidathion, Mevinphos, Omethoate, Parathion, Parathion-methyl, Phorate and Pirimiphos-methyl Profenofos.

Chromatographic analysis was performed by injecting 1 μL of solution into a GC system (mod. Trace 1310, Thermo Fisher Scientific, Lenexa, KS 66215, USA) coupled to an MS detector (mod. ISQ7000, Thermo Scientific, Lenexa, KS, USA) and managed by Chromeleon 7.2.10 software.

## 3. Results

### 3.1. Radiography

**Figure 2:**
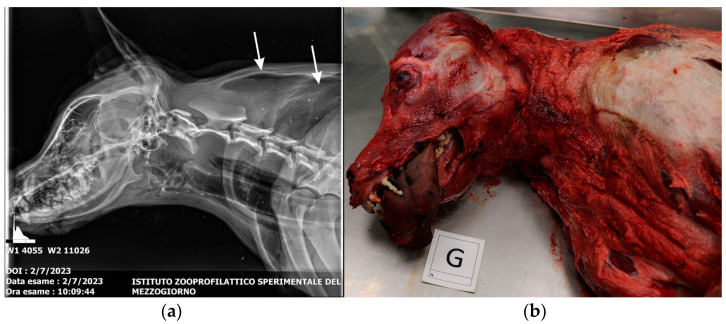
(**a**) Radiographic study of the skull and thorax in lateral projection, revealing the presence of pneumoderma of the neck (arrows), sternum and left scapular region; (**b**) skinning revealed gross lesions (letter G) associated with neck compression.

**Figure 3:**
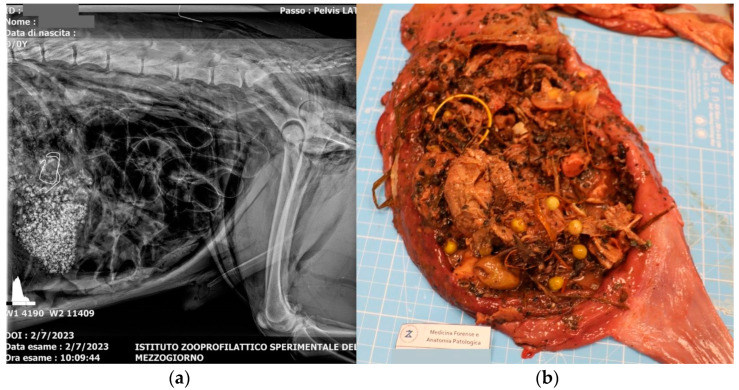
(**a**) Abdominal X-ray reveals the presence of granular and thread-like radiopaque material in the stomach; (**b**) the stomach content was traceable to food material (mushrooms, meat morsels, peas, bones, sausages, plastic, iron, olives and plant material) mixed with blackish microgranules, harmful substances (metal wire) and plastic material.

X-ray study of the skull and chest reveals pneumoderma of the neck, sternum and left scapular region (Figure 2a). In contrast, abdominal radiography reveals the presence of granular and thread-like radiopaque material in the stomach (Figure 3a).

### 3.2. External Examination and Necropsy

The external examination of the corpse revealed cutaneous hyperemia in the neck region, hemoptysis and congested mucous membranes. Skinning revealed the presence of hyperemia of the subcutaneous tissue in correspondence with the right and left forelimb, the muscles of the head and neck region (Figure 2b), the muscles of the abdominal region and the intercostal muscles.

The examination of the internal organs revealed blood in the trachea and a torn tracheal ring at the level of the cranial third, which, however, did not determine the opening of the tracheal lumen. Upon opening the thoracic cavity, the presence of moderate hemothorax was highlighted, and upon opening the heart, the presence of a small amount of uncoagulated blood was detected in the atrioventricular chambers. The gross examination of the lungs revealed increased volume and a dark-red color of the lobes, slightly increased consistency, moist cut surface and rich in dark blood. The stomach was full, and the contents were traceable to food material mixed with blackish microgranules, harmful substances (metal wire) and plastic material (Figure 3b).

### 3.3. Histology

**Figure 4:**
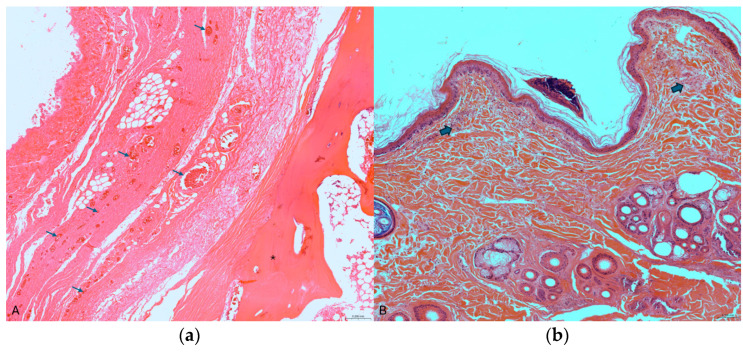
(**a**) Histological section of the hyoid bone (asterisk) showing multifocal areas of vascular congestion (arrows) (10×); (**b**) on the top of the image, an intracorneal pustule, subepidermal hemorrhagic infiltration and edema (arrows) (4×) (H&E).

Histopathological examination of the injured sections of the trachea showed extensive extravasation of erythrocytes in the peritracheal soft tissue and congestion of the blood vessels in the lamina propria [9]. In the hyoid bone, many small fragments were found associated with multifocal areas of vascular congestion in the surrounding soft tissue (Figure 4a) and disruption of the condral matrix of the laryngeal cartilage. In the site of the neck ligature mark, the skin showed hemorrhagic subepidermal infiltrate and moderate edema between the fibers of collagen. In this area, on the superficial epidermis, intracorneal pustules were also evident (Figure 4b), indicating cell damage related to the friction of a traumatic force. In the deep dermis, edema and blood effusion led to tissue disruption [9,10,11].

### 3.4. Toxicology

The pesticide test was negative; none of the studied compounds were detected in the gastric contents. However, the analysis of coumarin anticoagulants was positive, detecting the presence of second-generation 4-hydroxycoumarins, brodifacoum and difenacoum in the liver.

## 4. Discussion

To our knowledge, this is the first documented case of overkilling in a dog, which is defined as excessive injuries that exceed those necessary to cause the victim’s death. The difficulty is determining precisely how many injuries are necessary to define “overkill” in a specific homicide case.

The authors do not want to reduce the analysis of this phenomenon to the description of the number, type and distribution of wounds inflicted on the victim. The case report presented provides a detailed examination of a giant-breed male dog subjected to severe trauma and apparent poisoning. The findings highlight the critical role of forensic veterinary medicine in identifying and characterizing cases of “overkilling” in animals, a phenomenon rarely described in veterinary literature [12,13].

In this case, the extensive traumatic injuries and evidence of poisoning indicate a deliberate and violent act, consistent with the definition of overkilling. In particular, the presence of traumatic injuries, including skin petechiae, subcutaneous hemorrhage and a comminuted fracture of the hyoid bone, supports the conclusion of intentional strangulation. Furthermore, microscopic evaluation of pathological lesions in the skin, trachea and hyoid bone fully supports the diagnosis of intravital trauma [13].

Toxicology analysis detected second-generation anticoagulant rodenticides (brodifacoum and difenacoum) in the liver, while pesticide screening yielded negative results. These results suggest that poisoning was a secondary mechanism of injury, potentially intended to stun or kill the animal. The absence of detectable pesticides in the gastric contents but the presence of rodenticides in the liver indicates prior ingestion and systemic absorption of these toxic compounds. These substances are known to have potent anticoagulant properties, causing internal bleeding and exacerbating the physical trauma observed [14].

Radiographic imaging and autopsy provided critical insights. Pneumoderma, hemothorax and the presence of radiopaque materials in the stomach indicate a combination of external and internal injuries, potentially inflicted by mechanical asphyxiation and foreign body ingestion.

This case highlights the importance of recognizing animal abuse as a social problem with potential implications for public safety. The documented link between violence toward animals and interpersonal violence in humans reinforces the ethical and professional responsibility of veterinarians to identify and report such incidents [15,16]. Furthermore, integrating veterinary forensic practices with legal frameworks ensures accountability and justice in animal cruelty cases.

## 5. Conclusions

Future research should focus on standardizing the criteria for diagnosing overkill in veterinary settings and expanding the understanding of its implications. Collaboration between veterinary and human forensic experts could improve the ability to detect patterns of violence and prevent its escalation. This case contributes to the growing body of evidence supporting the role of forensic autopsy in documenting and addressing extreme acts of violence against animals for both ethical and legal purposes.

## Figures and Tables

**Table 1:** Mass spectrometric parameters of 6 rodenticides.

Compound	Precursor Ion (m/z)	Product Ion (m/z)
Brodifacoum	521	135–79
Bromadiolone	525	250–181
Coumachlor	341	284–161
Coumatetralyl	291	247–141
Difenacoum	443	293–135
Warfarin	307	250–161

## Data Availability

The original contributions presented in this study are included in the article.
